# Inappropriate claims from non-equivalent medications in osteoarthritis: a position paper endorsed by the European Society for Clinical and Economic Aspects of Osteoporosis, Osteoarthritis and Musculoskeletal Diseases (ESCEO)

**DOI:** 10.1007/s40520-017-0861-1

**Published:** 2017-11-24

**Authors:** Olivier Bruyère, Cyrus Cooper, Nasser M. Al-Daghri, Elaine M. Dennison, René Rizzoli, Jean-Yves Reginster

**Affiliations:** 1 Department of Public Health, Epidemiology and Health Economics, University of Liège, CHU Sart Tilman B23, 4000 Liège, Belgium; 2 MRC Lifecourse Epidemiology Unit, University of Southampton, Southampton General Hospital, Southampton, UK; 3 NIHR Musculoskeletal Biomedical Research Unit, University of Oxford, Oxford, UK; 4 Prince Mutaib Chair for Biomarkers of Osteoporosis, Biochemistry Department, College of Science, King Saud University, Riyadh, 11451 Saudi Arabia; 5 Division of Bone Diseases, Geneva University Hospitals and Faculty of Medicine, Geneva, Switzerland; 6 WHO Collaborating Centre for Public Health Aspects of Musculoskeletal Health and Aging, Liège, Belgium

**Keywords:** Chondroitin sulfate, Glucosamine, Symptomatic slow-acting drugs for osteoarthritis, Knee, Osteoarthritis

## Abstract

Osteoarthritis (OA) is a progressive joint disease, that occurs frequently in the aging population and is a major cause of disability worldwide. Both glucosamine and chondroitin are biologically active molecules that are substrates for proteoglycan, an essential component of the cartilage matrix. Evidence supports the use of glucosamine and chondroitin as symptomatic slow-acting drugs for osteoarthritis (SYSADOAs) with impact on OA symptoms and disease-modifying effects in the long term. Glucosamine and chondroitin are administered in exogenous form as a sulfate salt and multiple formulations of these agents are available, both as prescription-grade products and nutritional supplements. However, while all preparations may claim to deliver a therapeutic level of glucosamine or chondroitin not all are supported by clinical evidence. Only patented crystalline glucosamine sulfate (pCGS) is shown to deliver consistently high glucosamine bioavailability and plasma concentration in humans, which corresponds to demonstrated clinical efficacy. Similarly, clinical evidence supports only the pharmaceutical-grade chondroitin sulfate. The European Society for Clinical and Economic Aspects of Osteoporosis, Osteoarthritis and Musculoskeletal Diseases (ESCEO) advocates, through careful consideration of the evidence base, that judicious choice of glucosamine and chondroitin formulation is essential to maximize clinical benefit, patient adherence and satisfaction with treatment. In future, the ESCEO recommends that complex molecules with biological activity such as pCGS may be treated as “biosimilars” akin to the European Medicines Agency guidance on biological medicinal products. It seems likely that for all other complex molecules classed as SYSADOAs, the recommendation to use only formulations clearly supported by the evidence-base should apply.

**Electronic supplementary material:**

The online version of this article (10.1007/s40520-017-0861-1) contains supplementary material, which is available to authorized users.

## Introduction

Symptomatic slow-acting drugs for osteoarthritis (SYSADOAs) are a key component of the pharmacological treatment armamentarium for knee osteoarthritis (OA), a major cause of morbidity and disability in the aging population that may ultimately result in the need for joint replacement surgery [1–5]. Treatment guidelines from the European Society for Clinical and Economic Aspects of Osteoporosis, Osteoarthritis and Musculoskeletal Diseases (ESCEO) recommend the use of SYSADOAs as Step 1 pharmacological background therapy, with paracetamol as add-on rescue analgesia when needed [6]. However, there are many different agents in the class of SYSADOAs, including glucosamine, chondroitin, diacerein, and avocado soybean unsaponifiables, and not all are supported with a high level of clinical efficacy data, nor afforded with the same degree of recommendation in clinical guidelines [6–10].

Glucosamine and chondroitin occur naturally in the body as the principal substrates in the biosynthesis of proteoglycan, a compound essential for maintaining cartilage integrity. While both glucosamine and chondroitin have been developed as prescription drugs for OA, there are many products and formulations available as over-the-counter (OTC) medicines and dietary supplements, which all include various quantities of glucosamine and chondroitin. However, although these preparations claim to deliver a therapeutic level of glucosamine and chondroitin they are not supported by clinical evidence. By careful analysis of the available literature, in this paper, we will show that all preparations of glucosamine and chondroitin are not the same, and use of the incorrect formulations could result in sub-optimal outcomes, and consequently poor adherence and patient dissatisfaction with treatment. Hence, the ESCEO guidelines specifically recommend only prescription-grade glucosamine or chondroitin sulfate as guided by the evidence base [6].

## EMA guidance on “biosimilars”

Both glucosamine and chondroitin are biologically active molecules that occur in endogenous form as building blocks for complex long-chain glycosaminoglycans that make up part of the cartilage matrix. Chondroitin sulfate is a complex, heterogeneous polysaccharide extracted from various animal cartilages, and thus has a wide range of molecular weights and different amounts and patterns of sulfation [11]. Chondroitin sulfate is classified as a biological active substance by the European Medicines Agency (EMA) [12].

Although highly unstable in isolation, glucosamine may be delivered in exogenous form if carefully formulated as a stabilized oral delivery system that can deliver high bioavailability to the plasma, while avoiding extensive degradation by first-pass metabolism [13]. Due to its inherent instability and the special formulation of glucosamine required to deliver good bioavailability, differences in the molecular formulation of glucosamine preparations dictate a different approach to that of small molecule generic substitution. In this case, glucosamine may be considered in similar terms to the classification of a biological medicinal product as defined by the EMA. The EMA provides guidance on similar biological medicinal products—“biosimilars”—outlining the non-clinical and clinical requirements for a similar biological medicinal product [14]. A biosimilar is defined as a biological medicinal product that contains a version of the active substance of an already authorized original biological medicinal product (reference medicinal product). The nature and complexity of the reference product have an impact on the extent of the (non)clinical studies to confirm biosimilarity. The differences observed in the physicochemical and biological analyses will guide the planning of the (non-)clinical studies. Thus, to apply for marketing authorization for a biosimilar, the dossier will need to provide data demonstrating comparability with the reference medicinal product using appropriate physicochemical and in vitro biological tests, non-clinical studies and clinical studies.

## Glucosamine pharmacokinetics

Glucosamine sulfate (GS) is strongly hygroscopic and readily oxidized under normal conditions with the first signs of degradation appearing after 4 h and complete decomposition after 36 h [15]. To avoid stability problems most non-pharmaceutical grade GS products actually consist of glucosamine hydrochloride (GH) plus sodium sulfate. GH is the most readily available glucosamine salt found in many dietary supplements (Supplementary data: Fig. 1). The prescription, patented crystalline glucosamine sulfate (pCGS) formulation, on the other hand, consists of a mixed salt of glucosamine sulfate and sodium chloride as a crystalline powder with a melting point above 300 °C (Supplementary data: Fig. 1). pCGS is a uniquely stabilized form demonstrated to be perfectly preserved after 1 year at room temperature (25 °C) and 60% relative humidity (US Patent No. 4642340) [15].

The formulation of glucosamine in a stabilized delivery system is important to ensure maximized bioavailability of glucosamine in humans, measured as 44%, and high glucosamine concentration in plasma [13, 16, 17]. Pharmacokinetic studies show that administration of pCGS (1500 mg) leads to a mean plasma concentration at steady state of 9 µM in healthy volunteers [16]. In contrast, the peak plasma level of glucosamine reached after a single dose of GH (1500 mg), at 2.7 µM, is one-third of that measured with pCGS, while administration of GH at the dose routinely used in clinical practice (500 mg tid) leads to steady state levels of only 1.2 µM [18].

Although the mechanisms of action underlying the favourable actions of glucosamine are not yet fully elucidated, glucosamine is shown to induce reversal of the pro-inflammatory and joint-degenerating effects of interleukin-1 (IL-1) on osteoarthritic cartilage and chondrocytes [19, 20]. Studies in a human chondrocyte cell line show that pCGS inhibits the effect of IL-1beta (IL-1β), a potent pro-inflammatory cytokine, on the expression of inflammation markers and matrix degradation factors [21]. The maximal effect on human chondrocyte cells is achieved with a concentration of glucosamine in the 10 µM range, which corresponds to the magnitude of glucosamine concentration achieved in human plasma following administration of pCGS (1500 mg). Importantly, in OA patients, peak plasma glucosamine concentrations at 7.17 µM (range 3.35–22.7) have been measured after once-daily administration of pCGS (1500 mg) [17].

## Glucosamine efficacy

To assess the effect of formulation on glucosamine efficacy in knee OA we searched the literature for placebo-controlled trials and meta-analyses of glucosamine formulations, including GH, GS and pCGS. There are numerous studies published on the effect of glucosamine on the symptoms of knee OA, namely pain and functional impairment, giving a wide heterogeneity of results, larger than might be expected by chance [22]. Possible explanations given for the difference in efficacy found between trials have focused on the different glucosamine formulations, the poor quality of some trials, inadequate allocation concealment, and the potential risk of industry bias which may distort the results [22–24]. The Cochrane review of 25 RCTs of all glucosamine formulations in 4963 OA patients, when limited to trials with adequate concealment (11 RCTs) failed to show any benefit of glucosamine for pain [25]. However, when the trials using the pCGS formulation were analyzed in isolation, pCGS was found to be superior to placebo for pain [standardized mean difference (SMD) − 1.11; 95% confidence interval (CI) − 1.66 to − 0.57) and function (Lequesne index SMD − 0.47; 95% CI − 0.82 to − 0.12), while other glucosamine formulations failed to reach statistical significance for pain or function [25].

Eriksen et al. performed a stratified meta-analysis to address the potential risk of bias due to unsatisfactory handling of the data, i.e., during randomization and concealment and statistical analyses [24]. They found that only eight placebo-controlled studies met the standard for ‘low risk of bias’. This analysis confirmed that the five studies with glucosamine formulations excluding pCGS, even with a ‘low risk of bias’, found a non-significant effect on pain reduction (SMD 0.02; 95% CI − 0.08 to 0.12). In contrast, analysis of the three ‘low risk of bias’ studies with pCGS confirmed a reduction in pain with SMD effect size of − 0.27 (95% CI − 0.43 to − 0.12) indicating a beneficial, pain-reducing effect of pCGS compared with placebo [24, 26–28]. The findings of Eriksen et al. are in complete agreement with an earlier analysis of the same three trials of pCGS judged to be of highest quality using the Jadad quality score for clinical trials [23, 29]. The effect of pCGS on function was also measured in the three pivotal trials, with a pooled fixed-model effect size of 0.33 on WOMAC function (Western Ontario and McMaster Universities Osteoarthritis Index) (Table 1) [23].

**Table 1:** Symptom outcomes for patented crystalline glucosamine sulfate (pCGS) formulation after 6 months to 3 years of treatment for knee osteoarthritis. Table adapted from Reginster [23]

Outcome	Fixed-model effect size (95% CI)^a^
WOMAC scale	
Total	0.33 (0.17–0.49)
Pain	0.27 (0.12–0.43)
Function	0.33 (0.17–0.48)
Lequesne index^b^	0.38 (0.19–0.57)

^a^Pooled effect size: estimates and 95% CIs from fixed-model meta-analysis method using the pooled standard deviation in each study/outcome [26–28]

^b^Not assessed in one study [27]. Heterogeneity, *I* ^2^ = 0.00

A recent systematic review and individual patient data meta-analysis has sought to evaluate the efficacy of glucosamine in subgroups of people with hip and knee OA within predefined groups based upon pain severity, body mass index (BMI), sex, structural abnormalities and the presence of inflammation [30]. This analysis included data from 5 RCTs of 1625 patients with knee and hip OA, in which GS and GH formulations (*n* = 815) were compared with placebo (*n* = 810), representing 55% of all participants in RCTs of glucosamine versus placebo. The trials were defined as having low risk of bias and with low heterogeneity. Within each subgroup analyses, no significant differences were found for glucosamine over placebo for pain and function in the short- or long-term (at either 3 or 24 months). No data from the RCTs of pCGS were included in this analysis. The findings of this paper confirm earlier analyses showing that other non-pCGS preparations are ineffective in all patients [31].

Several factors may explain the difference in efficacy observed between quality clinical trials of glucosamine preparations. The superiority of pCGS may be explained by the unique stabilized formulation of glucosamine, single once-daily dosing regimen (1500 mg) and high bioavailability, reaching higher glucosamine concentration in the plasma, compared with other preparations [13, 32]. The effect size for pCGS on pain, estimated from analysis of placebo-controlled studies, may be considered as only moderate at 0.27 (SMD and fixed-model meta-analysis), but it is greater than that of paracetamol (with effect size from SMD of 0.14; 95% CI 0.05–0.22) [33], and in the same range as that achieved with a short course of oral non-steroidal anti-inflammatory drugs (NSAIDs) (SMD effect size = 0.29 95% CI 0.22–0.35) [34]. Oral NSAIDs are recommended as Step 2 treatment in persistently symptomatic OA patients [6]; however, concerns over their gastrointestinal and cardiovascular safety limit their use to intermittent or cyclical short-term treatment courses [35, 36]. Conversely, the effect of pCGS treatment on pain is demonstrated over 6 months to 3 years, without cause for safety concern, with an adverse event rate similar to that of placebo [26–28]. Evidence that pCGS affords a disease-modifying effect beyond symptom control in the long term is also provided by two trials that measured a delay in joint structure changes. Analysis of joint space width (JSW) at trial enrollment and after 3 years of treatment found a reduction in joint space narrowing (JSN) with pCGS. In one study, a significant difference in JSN of 0.33 mm (95% CI 0.12–0.54) was observed with pCGS versus placebo (*p* = 0.003) [27]. In the second study, pCGS treatment was shown to completely prevent narrowing of the joint (JSN + 0.04 mm; 95% CI − 0.06 to 0.14; *p* = 0.001) [28].

## Chondroitin sulfate

Commercially available chondroitin is a polymer of sulfated disaccharides of varying length and different patterns of sulfation (Supplementary data: Fig. 2). Chondroitin sulfate (CS) is, therefore, a complex, heterogeneous polysaccharide with varying charge density and molecular weight, which can affect its chemical properties and biological/pharmacological activities [11]. The bioavailability of chondroitin is around 10–20% [37]. Only the pharmaceutical grade CS has been evaluated for purity, content and physicochemical parameters, while dietary supplement preparations of CS are not subject to such strict regulations [38, 39].

As CS preparations can vary due to their origin, production and purification, so the biological effects of chondroitin sulfate preparations can also differ. CS has been reported to elicit anti-inflammatory effects, and increase in type II collagen and proteoglycans, a reduction in bone resorption and better anabolic/catabolic balance in chondrocytes [11]. Various meta-analyses of trial results have been conducted to assess whether CS has a beneficial effect on OA symptoms and disease progression with mixed findings, due in part to inclusion of studies using non-pharmaceutical grade CS [40–45].

For example, in one study employing dietary supplements of CS (800 mg) and/or GS (1500 mg) taken once-daily for 2 years, neither preparation alone or in combination was observed to have a significant benefit over placebo for pain in knee OA [46]. On the contrary, in studies that employed a well-characterized, prescription CS evidence of efficacy was found [47, 48]. Significant improvement in pain and function on the Lequesne index and visual analog scale (VAS) for pain were measured after 3 months’ treatment with CS as compared with placebo (*p* = 0.0001) [47]. A Cochrane review including 43 RCTs of 4962 participants treated with CS found a small to moderate benefit with an 8-point greater improvement in VAS pain score (range 0–100) and 2-point greater improvement in Lesquesne index (range 0–24) compared with placebo in studies up to 6 months, but mostly of low quality, with high heterogeneity between trials (*I* ^2^ = 70%) [49]. In one RCT of symptomatic knee OA, pharmaceutical-grade CS (800 mg) given daily for 6 months lead to improvement in pain and function with efficacy similar to that of the selective NSAID celecoxib (200 mg/day) [50].

CS is also demonstrated to have a beneficial effect on joint structure changes in patients with knee OA. A significant reduction in minimum joint space width (JSW) was shown in a study of 2-years’ treatment with CS (800 mg) compared with placebo (*p* < 0.0001), along with a significant reduction in WOMAC pain after 6 months (*p* < 0.05) [48].

Taken together, this evidence further confirms the concept of non-equivalence of biological preparations, and explains why the ESCEO specifically recommends only the prescription-grade CS and glucosamine preparations.

## ESCEO recommendations

While many studies are published on the use of SYSADOAs, the efficacy of this class, and notably of glucosamine and chondroitin, has been called into question largely due to inherent differences in the formulations employed in trials. It would appear that careless uninformed, and scientifically inaccurate analysis of the evidence base may still occur in the OA community [51], and a more considered approach to addressing the complexities of selected biologically active agents is required.

Among glucosamine preparations, only the prescription pCGS formulation is proven to be efficacious for the treatment of OA symptoms of pain and functional impairment, and may even offer some protection from disease progression in the long term. For all other glucosamine preparations, the evidence repeatedly demonstrates a minimal effect.

For CS, the available evidence points towards a similar conclusion: only the pharmaceutical-grade CS should be used for the treatment of knee OA. Thus, it is our opinion that from careful consideration of the evidence base, judicious choice of glucosamine and chondroitin formulation is essential to maximize treatment benefit.

In consideration of future research, we recommend that complex molecules with biological activity such as pCGS may be treated as “biosimilars” akin to EMA guidance for biological medicinal products, for which any other preparations must demonstrate comparability with the reference product in terms of physicochemical, in vitro, non-clinical and clinical studies, to be considered suitable for substitution [14].

In accordance with the 2010 European regulatory guideline on clinical investigation of medicinal products for OA, future clinical trials of SYSADOAs should measure the effect on symptom outcomes, pain and function, for a minimal duration of 6 months, and may determine structural changes over 2 years with JSN measurement. A placebo arm, and/or an active comparator arm must be included, as appropriate [52]. In addition, we recommend the effect of SYSADOAs on symptom outcomes should be measured at multiple time points over 6–12 months to reflect a sustained clinical benefit [53].

In the meantime, for current clinical practice, we recommend using only SYSADOA formulations with proven efficacy and safety data.

## Other SYSADOAs

It appears likely that for all other complex molecules classified as SYSADOAs, similar advice on the judicial use of only formulations clearly supported by the evidence-base should apply. For example, avocado soybean unsaponifiables (ASU) are natural vegetable extracts made from avocado and soybean oils. ASU is a complex mixture of many compounds including fat-soluble vitamins, sterols, triterpene alcohols, and possibly furan fatty acids. The identity of the active component(s) is unknown; however, ASU is shown to possess chondroprotective, anabolic and anti-catabolic properties [54]. In clinical studies, some improvement in WOMAC pain, stiffness and physical function have been demonstrated in the short-term with ASU (300 mg/day) [55–57], although mixed results for the effect of ASU on disease progression are found from studies of 2–3 years’ treatment in patients with hip or knee OA [58, 59].

## Conclusion

Through diligent review of the evidence base, we have demonstrated that informed selection of glucosamine and chondroitin formulation is essential to optimize treatment effect. Thus, the ESCEO guidelines specifically recommend only prescription-grade glucosamine or chondroitin sulfate to maximize clinical outcomes, while claims of equivalence from other formulations may be considered as inappropriate. In future, we recommend that complex molecules with biological activity such as pCGS may be treated as “biosimilars” corresponding to the EMA guidance on biological medicinal products, for which any other preparations must demonstrate comparability in terms of physicochemical, non-clinical and clinical characteristics. It seems likely that for all other complex molecules classed as SYSADOAs, the recommendation to use only formulations clearly supported by the evidence-base should equally apply.

## Electronic supplementary material

Below is the link to the electronic supplementary material.

**Fig. 1** The chemical structure of glucosamine and its salts. MW, molecular weight (PPTX 51 KB)

**Fig. 2** The chemical structure of chondroitin sulfate. The chemical structure identifies one unit in a chondroitin sulfate chain. A chondroitin chain can have over 100 individual sugars, each of which can be sulfated in variable positions and quantities. For example, Chondroitin-4-sulfate: R_1_ = H; R_2_ = SO_3_H; R_3_ = H; Chondroitin-6-sulfate: R_1_ = SO_3_H; R_2_, R_3_ = H. (PPTX 65 KB)
