# Supplementary material for: Inappropriate claims from non-equivalent medications in osteoarthritis: a position paper endorsed by the European Society for Clinical and Economic Aspects of Osteoporosis, Osteoarthritis and Musculoskeletal Diseases (ESCEO)
Source: Aging Clin Exp Res. 2017 Nov 24;30(2):111–7. doi: 10.1007/s40520-017-0861-1 (PMC5814472; doi:10.1007/s40520-017-0861-1)
Supplement: Supplementary file 1 — Fig. 1 The chemical structure of glucosamine and its salts. MW, molecular weight (PPTX 51 KB) [file 40520_2017_861_MOESM1_ESM.pptx]

## Slide 1
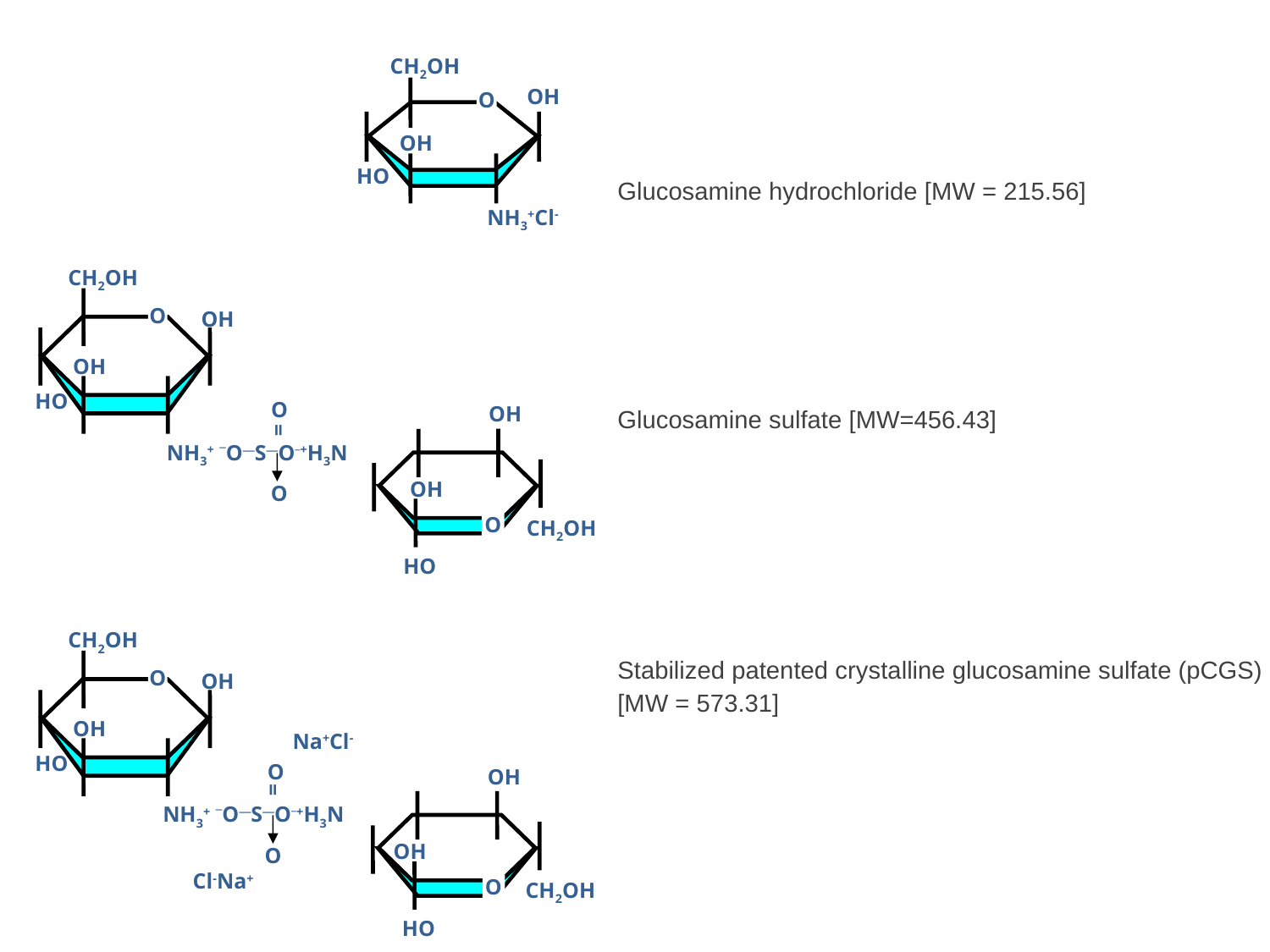

CH2OH
OH
O
OH
HO
NH3+Cl-
Glucosamine hydrochloride [MW = 215.56]
CH2OH
O
OH
OH
HO
O
OH
=
NH3+ _O__S__O_+H3N
OH
O
CH2OH
O
HO
Glucosamine sulfate [MW=456.43]
CH2OH
O
OH
OH
Na+Cl-
HO
O
OH
=
NH3+ _O__S__O_+H3N
OH
 O
Cl-Na+
CH2OH
O
HO
Stabilized patented crystalline glucosamine sulfate (pCGS) [MW = 573.31]
