# Supplementary figures and images for: Inappropriate claims from non-equivalent medications in osteoarthritis: a position paper endorsed by the European Society for Clinical and Economic Aspects of Osteoporosis, Osteoarthritis and Musculoskeletal Diseases (ESCEO)
Source: Aging Clin Exp Res. 2017 Nov 24;30(2):111–7. doi: 10.1007/s40520-017-0861-1 (PMC5814472; doi:10.1007/s40520-017-0861-1)

## Slide 1
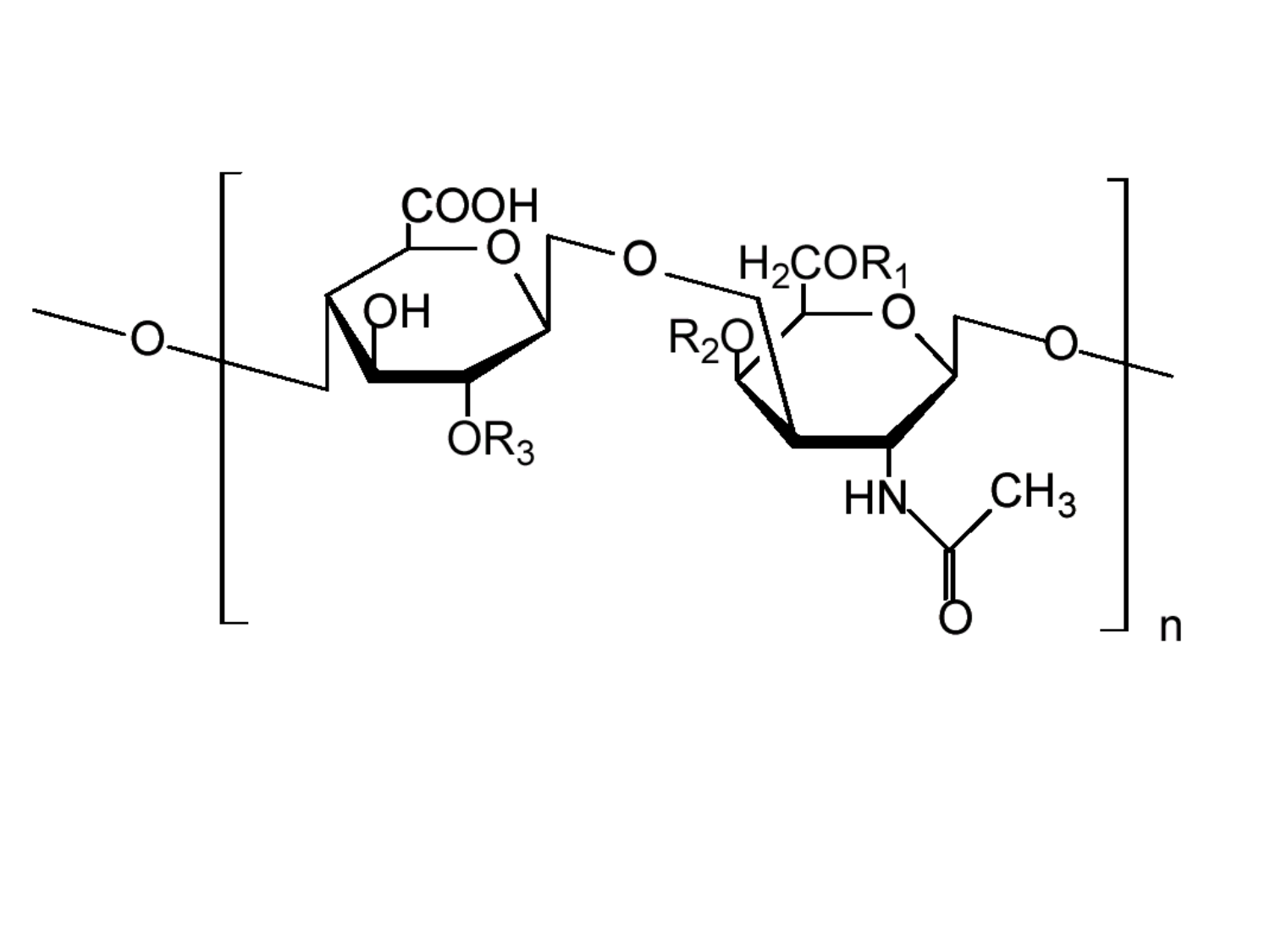

#

Supplement: Supplementary file 2 — Fig. 2 The chemical structure of chondroitin sulfate. The chemical structure identifies one unit in a chondroitin sulfate chain. A chondroitin chain can have over 100 individual sugars, each of which can be sulfated in variable positions and quantities. For example, Chondroitin-4-sulfate: R1 = H; R2 = SO3H; R3 = H; Chondroitin-6-sulfate: R1 = SO3H; R2, R3 = H. (PPTX 65 KB) [file 40520_2017_861_MOESM2_ESM.pptx]
